# Ring-expansion synthesis and crystal structure of dimethyl 4-ethyl-1,4,5,6,7,8-hexa­hydro­azonino[5,6-*b*]indole-2,3-di­carboxyl­ate

**DOI:** 10.1107/S205698901700161X

**Published:** 2017-02-07

**Authors:** Van Tuyen Nguyen, Elena A. Sorokina, Anna V. Listratova, Leonid G. Voskressensky, Nikolai N. Lobanov, Pavel V. Dorovatovskii, Yan V. Zubavichus, Victor N. Khrustalev

**Affiliations:** a Institute of Chemistry, Vietnam Academy of Science and Technology, 18 Hoang Quoc Viet, Hanoi, Vietnam; b Graduate University of Science and Technology, 18 Hoang Quoc Viet, Hanoi, Vietnam; c Organic Chemistry Department, Peoples’ Friendship University of Russia, 6 Miklukho-Maklaya St., Moscow 117198, Russian Federation; d Inorganic Chemistry Department, Peoples’ Friendship University of Russia, 6 Miklukho-Maklaya St., Moscow 117198, Russian Federation; e National Research Centre "Kurchatov Institute", 1 Acad. Kurchatov Sq., Moscow 123182, Russian Federation; f Inorganic Chemistry Department, Peoples’ Friendship University of Russia, 6 Miklukho-Maklay St., Moscow 117198, Russian Federation; g X-Ray Structural Centre, A.N. Nesmeyanov Institute of Organoelement Compounds, Russian Academy of Sciences, 28 Vavilov St., B–334, Moscow 119991, Russian Federation

**Keywords:** crystal structure, synchrotron radiation, natural alkaloids, azonino­indoles, Alz­heim­er’s disease

## Abstract

The title compound, C_20_H_24_N_2_O_4_, is the product of a ring-expansion reaction from a seven-membered hexa­hydro­azepine to a nine-membered azonine. The azonine ring of the mol­ecule adopts a chair–boat conformation. In the crystal, mol­ecules are linked by bifurcated N—H⋯(O,O) hydrogen bonds, generating [010] zigzag chains. The title compound shows inhibitory activity against acetyl­cholinesterase and butyrylcholinesterase, and might be considered as a candidate for the design of new types of anti-Alzheimer’s drugs.

## Abstract 2

The nine-membered azonino­indole ring in the title compound arose from a ring-expansion reaction. The title compound shows acetyl­cholinesterase and butyrylcholinesterase inhibition.

## Chemical context   

The azonine moiety has long been known as a building block of natural alkaloids (Neuss *et al.*, 1959 ▸, 1962 ▸; Uprety & Bhakuni, 1975 ▸). Azonine derivatives are known to act as ligands towards different receptors, thus demonstrating diverse types of biological activity (Magnus *et al.*, 1987 ▸; Kuehne, Bornman *et al.*, 2003 ▸; Kuehne, He *et al.*, 2003 ▸; Afsah *et al.*, 2009 ▸; Rostom, 2010 ▸; Tanaka *et al.*, 2014 ▸; Soldi *et al.*, 2015 ▸; Hartman & Kuduk, 2016 ▸).

The direct synthesis of such systems from acyclic precursors is difficult due to thermodynamic and kinetic limitations and hence the search for novel and efficient synthetic routes to medium-sized rings has attracted appreciable attention in recent years. Earlier, we elaborated a ring-expansion reaction from a six-membered tetra­hydro­pyridine ring to an eight-membered azocine ring under the action of activated alkynes applicable to fused tetra­hydro­pyridines (Voskressensky *et al.*, 2004 ▸; Voskressensky, Borisova *et al.*, 2006 ▸).

**Figure 1:**
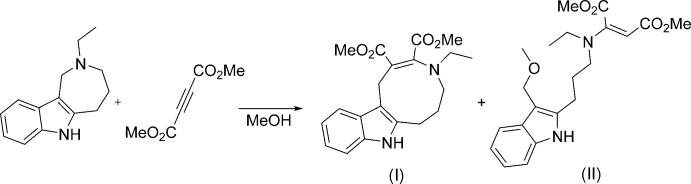
The synthesis of dimethyl 4-ethyl-1,4,5,6,7,8-hexa­hydro­azonino[5,6-*b*]indole-2,3-di­carboxyl­ate, (I), in methanol.

Herewith, we report on the synthesis of nine-membered azonine ring from a seven-membered hexa­hydro­azepine precursor using a similar reaction. More specifically, the initial 2-ethyl-1,2,3,4,5,6-hexa­hydro­azepino[4,3-*b*]indole in a methanol solution at room temperature under the action of dimethyl acetyl­enedi­carboxyl­ate undergoes a series of tandem transformations involving the hexa­hydro­azepine ring giving rise to azonino­indole (I) and 3-meth­oxy­methyl-substituted indole (II) (Fig. 1 ▸).

The title compound (I) has been tested *in vitro* for acetyl­cholinesterase and butyrylcholinesterase inhibition and demonstrated the inhibitor activity of 33.1 µ*M* and 89.1 µ*M* against acetyl­cholinesterase and butyrylcholinesterase, respectively. Thus, azonino­indoles might be considered as candidates for the design of new types of anti-Alzheimer’s drugs.
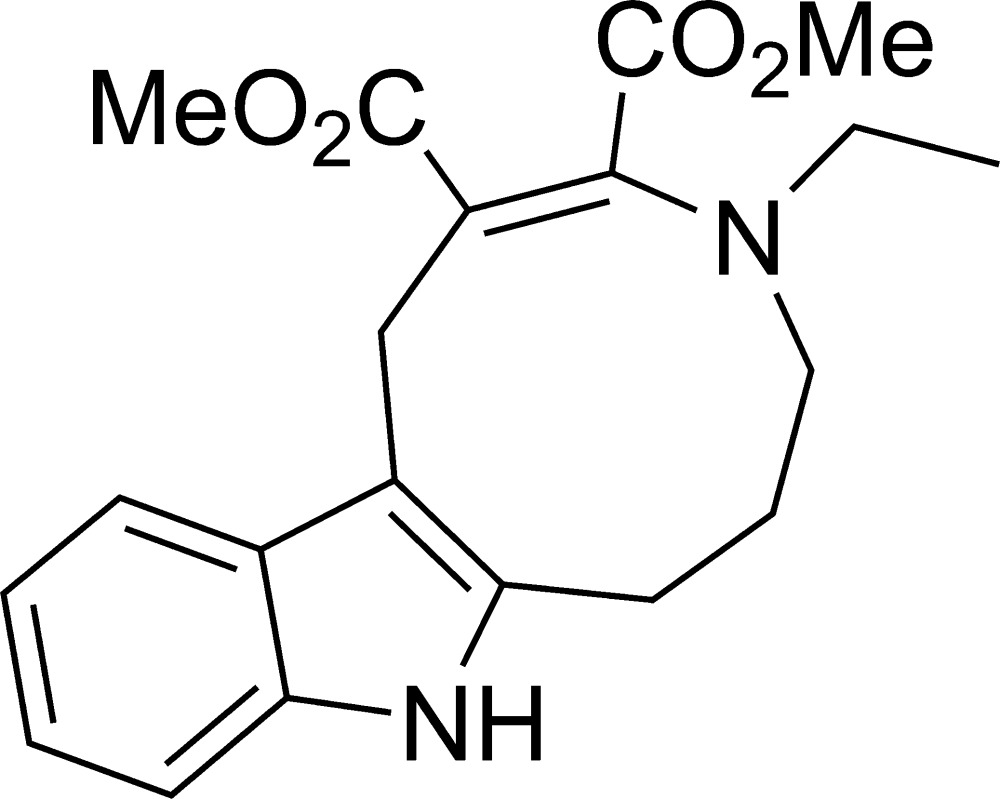


## Structural commentary   

**Figure 2:**
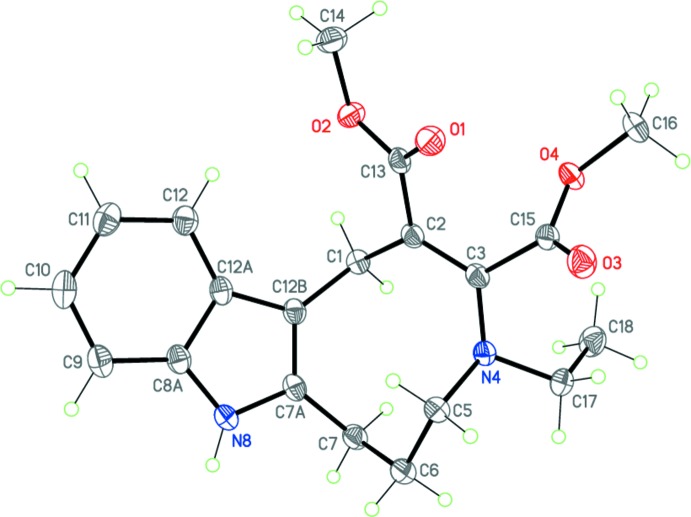
The mol­ecular structure of (I). Displacement ellipsoids are drawn at the 50% probability level. H atoms are shown as small spheres of arbitrary radius.

The title compound (I) is the product of the ring expansion described above. Its mol­ecular structure is unambiguously confirmed by the X-ray diffraction study (Fig. 2 ▸).

The nine-membered azonine ring of the mol­ecule adopts a chair–boat conformation (the basal planes are C5–C6/C7*A*–C12*B* and N4–C5/C1–C12*B*, respectively). It should be noted that the analogous nine-membered azonine ring in the related compound methyl 4-ethyl-11-methyl-1,4,5,6,7,8-hexa­hydro­azonino [5,6-*b*]indole-2-carboxyl­ate adopts a twisted *boat* conformation (Voskressensky, Akbulatov *et al.*, 2006 ▸). The C2=C3 and C3—N4 bond lengths [1.361 (2) and 1.401 (2) Å, respectively] in (I) indicate the presence of conjugation within the enamine C2=C3—N4 fragment. The substituent planes at the C2=C3 double bond are twisted by 18.12 (13)°, presumably due to steric reasons. The N4 nitro­gen atom has a trigonal-pyramidal configuration (sum of the bond angles is 345.5°). The inter­planar angle between the carboxyl­ate substituents is 59.74 (6)°.

## Supra­molecular features   

**Figure 3:**
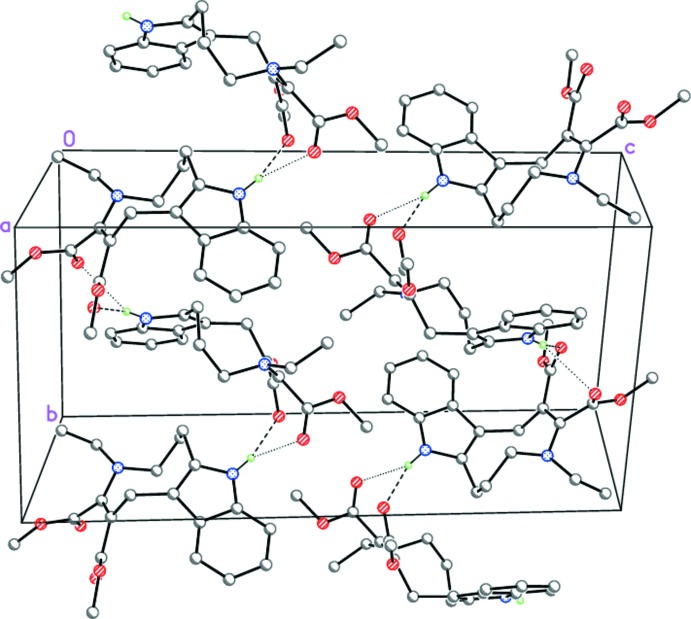
The crystal packing of (I), viewed along the crystallographic *a* axis. Dashed and dotted lines indicate the bifurcated N—H⋯(O,O) hydrogen bonds.

In the crystal, mol­ecules of (I) form zigzag chains propagating in the [010] direction by bifurcated N—H⋯(O,O) hydrogen-bonding inter­actions (Table 1 ▸) which are further packed in stacks toward [100] (Fig. 3 ▸).

## Synthesis and crystallization   

Dimethyl acetyl­enedi­carboxyl­ate (170 mg, 1.2 mmol) was added to 2-ethyl-1,2,3,4,5,6-hexa­hydro­azepino[4,3-*b*]indole (214 mg, 1 mmol) dissolved in methanol (10 ml). The reaction mixture was stirred for 2 h at room temperature and the progress of the reaction monitored by TLC. Then, the solvent was removed *in vacuo* and the residue was chromatographed over silica with ethyl­acetate:hexane as eluent to yield the target azonino­indole (I) (23%) and 3-meth­oxy­methyl­indole (II). Colourless prisms of (I) were grown by slow evaporation of an ethyl­acetate:hexane solution, m.p. 428–430 K. NMR ^1^H [CDCl_3_, δ (ppm), *J* (Hz)]: 1.03 (*t*, 3H, *J* = 7.2, CH_3_CH_2_), 1.77 (*m*, 2H, 6-CH_2_), 2.76 (*q*, 2H, *J* = 7.2, CH_3_CH_2_), 2.83 (*m*, 2H, 7-CH_2_), 3.08 (*m*, 2H, 5-CH_2_), 4.03 (*s*, 2H, 1-CH_2_), 3.75 (*s*, 3H, CO_2_CH_3_), 3.77 (*s*, 3H, CO_2_CH_3_), 7.09 (*m*, 2H, CH), 7.26 (*d*, 1H, *J* = 7.6, CH), 7.50 (*d*, 1H, *J* = 7.6, CH), 7.83 (*br.s*, 1H, NH). NMR ^13^C [DMSO-*d*_6_, δ (ppm), *J* (Hz)]: 15.2 (CH_3_), 22.6 (CH_2_), 24.0 (CH_2_), 27.0 (CH_2_), 44.5 (CH_2_), 52.2 (CH_3_), 52.3 (CH_3_), 55.6 (CH_2_), 108.3 (C), 111.0 (CH), 117.8 (CH), 118.7 (CH), 120.5 (CH), 124.4 (**?**), 128.1 (C), 135.3 (C), 135.6 (C), 151.1 (C), 166.3 (C), 169.3 (C). IR (KBr): *ν* (cm^−1^) = 1670, 3379. Found (%): C, 67.40; H, 6.79; N, 7.86. C_20_H_24_N_2_O_4_. Calculated (%): C, 67.30; H, 7.06; N, 8.00. Mass-spectrometry, *m*/*z* [*I*_rel_(%)]: 356 [*M* ^+^] (60), 327 (10), 297 (60), 267 (30), 252 (10), 237 (30), 226 (10), 209 (20), 180 (30), 168 (40), 156 (60), 143 (45), 128 (20), 115 (20), 77 (10), 58 (100), 45 (30).

## Refinement   

Crystal data, data collection and structure refinement details are summarized in Table 2 ▸. The amino-H atom was localized in Fourier syntheses and its position freely refined. The C-bound H atoms were placed in calculated positions with C—H = 0.95 Å (aryl-H), 0.96 Å (methyl-H), and 0.98 Å (methyl­ene-H) and refined in the riding-model approximation with the constraint *U*_iso_(H) = 1.5*U*_eq_(C) for the methyl groups and 1.2*U*_eq_(C or N) for all other H atoms.

## Supplementary Material

Crystal structure: contains datablock(s) global, I. DOI: 10.1107/S205698901700161X/hb7645sup1.cif

Structure factors: contains datablock(s) I. DOI: 10.1107/S205698901700161X/hb7645Isup2.hkl

Supporting information file. DOI: 10.1107/S205698901700161X/hb7645Isup3.cml

CCDC reference: 1530378

Additional supporting information:  crystallographic information; 3D view; checkCIF report

## Figures and Tables

**Table 1:** Hydrogen-bond geometry (Å, °)

*D*—H⋯*A*	*D*—H	H⋯*A*	*D*⋯*A*	*D*—H⋯*A*
N8—H8⋯O1^i^	0.891 (17)	2.234 (18)	3.0690 (18)	155.7 (14)
N8—H8⋯O3^i^	0.891 (17)	2.546 (16)	3.1029 (16)	121.2 (12)

Symmetry code: (i) 
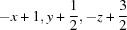
.

**Table 2:** Experimental details

Crystal data	
Chemical formula	C_20_H_24_N_2_O_4_
*M*_r_	356.41
Crystal system, space group	Monoclinic, *P*2_1_/*c*
Temperature (K)	100
*a*, *b*, *c* (Å)	8.5900 (17), 10.450 (2), 20.670 (4)
β (°)	98.45 (3)
*V* (Å^3^)	1835.3 (6)
*Z*	4
Radiation type	Synchrotron, λ = 0.96990 Å
μ (mm^−1^)	0.19
Crystal size (mm)	0.20 × 0.08 × 0.05

Data collection	
Diffractometer	Rayonix SX165 CCD
Absorption correction	Multi-scan (*SCALA*; Evans, 2006 ▸)
*T*_min_, *T*_max_	0.960, 0.990
No. of measured, independent and observed [*I* > 2σ(*I*)] reflections	20559, 3894, 3123
*R*_int_	0.068
(sin θ/λ)_max_ (Å^−1^)	0.641

Refinement	
*R*[*F* ^2^ > 2σ(*F* ^2^)], *wR*(*F* ^2^), *S*	0.046, 0.122, 1.01
No. of reflections	3894
No. of parameters	242
H-atom treatment	H atoms treated by a mixture of independent and constrained refinement
Δρ_max_, Δρ_min_ (e Å^−3^)	0.34, −0.24

Computer programs: *Automar* (MarXperts, 2015 ▸), *iMosflm* (Battye *et al.*, 2011 ▸) and *SHELXTL* (Sheldrick, 2015 ▸).

## supplementary crystallographic information

### Crystal data

**Table:**

C_20_H_24_N_2_O_4_	*F*(000) = 760
*M**_r_* = 356.41	*D*_x_ = 1.290 Mg m^−^^3^
Monoclinic, *P*2_1_/*c*	Synchrotron radiation, λ = 0.96990 Å
*a* = 8.5900 (17) Å	Cell parameters from 600 reflections
*b* = 10.450 (2) Å	θ = 3.8–38.0°
*c* = 20.670 (4) Å	µ = 0.19 mm^−^^1^
β = 98.45 (3)°	*T* = 100 K
*V* = 1835.3 (6) Å^3^	Prism, colourless
*Z* = 4	0.20 × 0.08 × 0.05 mm

### Data collection

**Table:**

Rayonix SX165 CCD diffractometer	3123 reflections with *I* > 2σ(*I*)
/f scan	*R*_int_ = 0.068
Absorption correction: multi-scan (*Scala*; Evans, 2006)	θ_max_ = 38.5°, θ_min_ = 3.8°
*T*_min_ = 0.960, *T*_max_ = 0.990	*h* = −10→10
20559 measured reflections	*k* = −13→12
3894 independent reflections	*l* = −26→26

### Refinement

**Table:**

Refinement on *F*^2^	Secondary atom site location: difference Fourier map
Least-squares matrix: full	Hydrogen site location: mixed
*R*[*F*^2^ > 2σ(*F*^2^)] = 0.046	H atoms treated by a mixture of independent and constrained refinement
*wR*(*F*^2^) = 0.122	*w* = 1/[σ^2^(*F*_o_^2^) + (0.0484*P*)^2^ + 0.484*P*] where *P* = (*F*_o_^2^ + 2*F*_c_^2^)/3
*S* = 1.01	(Δ/σ)_max_ = 0.001
3894 reflections	Δρ_max_ = 0.34 e Å^−^^3^
242 parameters	Δρ_min_ = −0.24 e Å^−^^3^
0 restraints	Extinction correction: SHELXL, Fc^*^=kFc[1+0.001xFc^2^λ^3^/sin(2θ)]^-1/4^
Primary atom site location: difference Fourier map	Extinction coefficient: 0.0139 (16)

### Special details

**Table:**

Geometry. All esds (except the esd in the dihedral angle between two l.s. planes) are estimated using the full covariance matrix. The cell esds are taken into account individually in the estimation of esds in distances, angles and torsion angles; correlations between esds in cell parameters are only used when they are defined by crystal symmetry. An approximate (isotropic) treatment of cell esds is used for estimating esds involving l.s. planes.

### Fractional atomic coordinates and isotropic or equivalent isotropic displacement parameters (Å^2^)

**Table:**

	*x*	*y*	*z*	*U*_iso_*/*U*_eq_	
O1	0.63858 (11)	0.12498 (9)	0.59679 (5)	0.0243 (3)	
O2	0.85193 (11)	0.25114 (9)	0.61630 (4)	0.0223 (3)	
O3	0.27706 (12)	0.17391 (10)	0.54825 (5)	0.0265 (3)	
O4	0.45470 (11)	0.26071 (9)	0.49055 (4)	0.0224 (3)	
C1	0.68135 (16)	0.46000 (13)	0.65876 (6)	0.0200 (3)	
H1A	0.7916	0.4641	0.6501	0.024*	
H1B	0.6278	0.5385	0.6402	0.024*	
C2	0.60249 (16)	0.34538 (13)	0.62131 (6)	0.0186 (3)	
C3	0.44674 (16)	0.34394 (13)	0.59639 (6)	0.0187 (3)	
N4	0.33815 (13)	0.42675 (11)	0.61889 (5)	0.0201 (3)	
C5	0.30356 (16)	0.39814 (14)	0.68573 (6)	0.0217 (3)	
H5A	0.3899	0.3452	0.7088	0.026*	
H5B	0.2055	0.3471	0.6821	0.026*	
C6	0.28472 (17)	0.51732 (14)	0.72650 (6)	0.0245 (3)	
H6A	0.2598	0.4909	0.7698	0.029*	
H6B	0.1950	0.5684	0.7046	0.029*	
C7	0.43311 (17)	0.60151 (14)	0.73627 (6)	0.0240 (3)	
H7A	0.4487	0.6391	0.6937	0.029*	
H7B	0.4179	0.6726	0.7664	0.029*	
C7A	0.57725 (16)	0.52745 (13)	0.76366 (6)	0.0210 (3)	
N8	0.61170 (14)	0.50489 (12)	0.83032 (5)	0.0225 (3)	
H8	0.5647 (19)	0.5452 (16)	0.8602 (8)	0.027*	
C8A	0.74364 (16)	0.42812 (14)	0.84258 (6)	0.0216 (3)	
C9	0.82348 (17)	0.38193 (14)	0.90185 (6)	0.0256 (3)	
H9	0.7891	0.4019	0.9423	0.031*	
C10	0.95495 (18)	0.30578 (15)	0.89926 (7)	0.0287 (4)	
H10	1.0110	0.2723	0.9387	0.034*	
C11	1.00664 (17)	0.27727 (15)	0.83940 (7)	0.0274 (3)	
H11	1.0976	0.2256	0.8391	0.033*	
C12	0.92688 (17)	0.32340 (14)	0.78055 (7)	0.0237 (3)	
H12	0.9627	0.3035	0.7404	0.028*	
C12A	0.79257 (16)	0.39984 (13)	0.78146 (6)	0.0200 (3)	
C12B	0.68382 (16)	0.46324 (13)	0.73182 (6)	0.0196 (3)	
C13	0.69611 (15)	0.23057 (13)	0.60961 (6)	0.0188 (3)	
C14	0.94677 (17)	0.13776 (14)	0.60964 (7)	0.0262 (3)	
H14A	0.9175	0.1012	0.5659	0.039*	
H14B	0.9282	0.0744	0.6426	0.039*	
H14C	1.0584	0.1613	0.6159	0.039*	
C15	0.38326 (16)	0.24798 (13)	0.54379 (6)	0.0196 (3)	
C16	0.41346 (18)	0.16510 (15)	0.44026 (7)	0.0284 (4)	
H16A	0.4619	0.1872	0.4017	0.043*	
H16B	0.2988	0.1622	0.4283	0.043*	
H16C	0.4517	0.0812	0.4568	0.043*	
C17	0.19643 (16)	0.46212 (14)	0.57286 (6)	0.0230 (3)	
H17A	0.1268	0.5155	0.5959	0.028*	
H17B	0.1380	0.3835	0.5575	0.028*	
C18	0.23772 (18)	0.53519 (15)	0.51430 (7)	0.0291 (4)	
H18A	0.1409	0.5590	0.4856	0.044*	
H18B	0.3025	0.4811	0.4901	0.044*	
H18C	0.2965	0.6127	0.5293	0.044*	

### Atomic displacement parameters (Å^2^)

**Table:**

	*U*^11^	*U*^22^	*U*^33^	*U*^12^	*U*^13^	*U*^23^
O1	0.0240 (6)	0.0204 (6)	0.0279 (5)	−0.0006 (4)	0.0022 (4)	−0.0029 (4)
O2	0.0184 (5)	0.0221 (6)	0.0265 (5)	0.0014 (4)	0.0033 (4)	−0.0013 (4)
O3	0.0249 (6)	0.0291 (6)	0.0250 (5)	−0.0061 (4)	0.0027 (4)	−0.0020 (4)
O4	0.0268 (6)	0.0254 (6)	0.0151 (4)	−0.0018 (4)	0.0037 (4)	−0.0034 (4)
C1	0.0205 (7)	0.0204 (7)	0.0190 (6)	−0.0003 (5)	0.0024 (5)	−0.0005 (5)
C2	0.0210 (7)	0.0206 (7)	0.0143 (6)	0.0001 (5)	0.0027 (5)	0.0003 (5)
C3	0.0230 (7)	0.0186 (7)	0.0144 (6)	0.0011 (5)	0.0026 (5)	0.0003 (5)
N4	0.0205 (6)	0.0236 (7)	0.0157 (5)	0.0033 (5)	0.0007 (4)	0.0000 (4)
C5	0.0217 (7)	0.0253 (8)	0.0181 (6)	0.0012 (6)	0.0036 (5)	−0.0002 (5)
C6	0.0252 (8)	0.0287 (8)	0.0196 (6)	0.0047 (6)	0.0030 (5)	−0.0025 (6)
C7	0.0279 (8)	0.0237 (8)	0.0199 (6)	0.0047 (6)	0.0024 (5)	−0.0035 (5)
C7A	0.0259 (8)	0.0192 (7)	0.0172 (6)	−0.0017 (6)	0.0012 (5)	−0.0020 (5)
N8	0.0248 (7)	0.0265 (7)	0.0161 (5)	0.0015 (5)	0.0024 (5)	−0.0031 (5)
C8A	0.0234 (7)	0.0213 (7)	0.0194 (6)	−0.0045 (6)	0.0005 (5)	−0.0005 (5)
C9	0.0273 (8)	0.0294 (9)	0.0190 (6)	−0.0067 (6)	0.0003 (5)	0.0009 (6)
C10	0.0297 (8)	0.0283 (8)	0.0248 (7)	−0.0048 (6)	−0.0066 (6)	0.0052 (6)
C11	0.0237 (8)	0.0237 (8)	0.0324 (8)	−0.0006 (6)	−0.0033 (6)	0.0004 (6)
C12	0.0230 (8)	0.0232 (8)	0.0240 (6)	−0.0033 (6)	0.0000 (5)	−0.0022 (5)
C12A	0.0218 (7)	0.0181 (7)	0.0190 (6)	−0.0053 (5)	−0.0010 (5)	−0.0014 (5)
C12B	0.0212 (7)	0.0182 (7)	0.0187 (6)	−0.0017 (5)	0.0004 (5)	−0.0018 (5)
C13	0.0210 (7)	0.0226 (8)	0.0126 (6)	−0.0007 (5)	0.0015 (5)	0.0008 (5)
C14	0.0235 (8)	0.0254 (8)	0.0301 (7)	0.0049 (6)	0.0051 (6)	−0.0011 (6)
C15	0.0204 (7)	0.0219 (7)	0.0158 (6)	0.0034 (5)	0.0006 (5)	0.0008 (5)
C16	0.0307 (8)	0.0338 (9)	0.0195 (6)	−0.0007 (7)	0.0004 (6)	−0.0102 (6)
C17	0.0203 (7)	0.0265 (8)	0.0210 (6)	0.0032 (6)	−0.0011 (5)	0.0013 (5)
C18	0.0276 (8)	0.0320 (9)	0.0266 (7)	0.0043 (6)	−0.0001 (6)	0.0070 (6)

### Geometric parameters (Å, º)

**Table:**

O1—C13	1.2221 (16)	C7A—N8	1.3862 (16)
O2—C13	1.3426 (17)	N8—C8A	1.3813 (19)
O2—C14	1.4558 (17)	N8—H8	0.891 (17)
O3—C15	1.2101 (17)	C8A—C9	1.3991 (18)
O4—C15	1.3431 (17)	C8A—C12A	1.4198 (19)
O4—C16	1.4478 (16)	C9—C10	1.389 (2)
C1—C12B	1.5077 (18)	C9—H9	0.9500
C1—C2	1.5292 (18)	C10—C11	1.407 (2)
C1—H1A	0.9900	C10—H10	0.9500
C1—H1B	0.9900	C11—C12	1.3916 (19)
C2—C3	1.3612 (19)	C11—H11	0.9500
C2—C13	1.4838 (19)	C12—C12A	1.406 (2)
C3—N4	1.4010 (18)	C12—H12	0.9500
C3—C15	1.5201 (18)	C12A—C12B	1.4432 (18)
N4—C17	1.4776 (16)	C14—H14A	0.9800
N4—C5	1.4857 (17)	C14—H14B	0.9800
C5—C6	1.526 (2)	C14—H14C	0.9800
C5—H5A	0.9900	C16—H16A	0.9800
C5—H5B	0.9900	C16—H16B	0.9800
C6—C7	1.537 (2)	C16—H16C	0.9800
C6—H6A	0.9900	C17—C18	1.517 (2)
C6—H6B	0.9900	C17—H17A	0.9900
C7—C7A	1.4988 (19)	C17—H17B	0.9900
C7—H7A	0.9900	C18—H18A	0.9800
C7—H7B	0.9900	C18—H18B	0.9800
C7A—C12B	1.378 (2)	C18—H18C	0.9800
			
C13—O2—C14	115.04 (11)	C8A—C9—H9	121.3
C15—O4—C16	115.27 (11)	C9—C10—C11	121.25 (13)
C12B—C1—C2	117.68 (11)	C9—C10—H10	119.4
C12B—C1—H1A	107.9	C11—C10—H10	119.4
C2—C1—H1A	107.9	C12—C11—C10	121.14 (14)
C12B—C1—H1B	107.9	C12—C11—H11	119.4
C2—C1—H1B	107.9	C10—C11—H11	119.4
H1A—C1—H1B	107.2	C11—C12—C12A	119.00 (14)
C3—C2—C13	117.09 (12)	C11—C12—H12	120.5
C3—C2—C1	122.54 (13)	C12A—C12—H12	120.5
C13—C2—C1	120.35 (11)	C12—C12A—C8A	118.76 (12)
C2—C3—N4	122.20 (12)	C12—C12A—C12B	134.28 (13)
C2—C3—C15	120.48 (12)	C8A—C12A—C12B	106.96 (12)
N4—C3—C15	117.29 (11)	C7A—C12B—C12A	106.86 (11)
C3—N4—C17	117.81 (11)	C7A—C12B—C1	125.23 (12)
C3—N4—C5	114.73 (11)	C12A—C12B—C1	127.89 (13)
C17—N4—C5	113.00 (11)	O1—C13—O2	122.15 (13)
N4—C5—C6	113.66 (12)	O1—C13—C2	123.62 (12)
N4—C5—H5A	108.8	O2—C13—C2	114.19 (12)
C6—C5—H5A	108.8	O2—C14—H14A	109.5
N4—C5—H5B	108.8	O2—C14—H14B	109.5
C6—C5—H5B	108.8	H14A—C14—H14B	109.5
H5A—C5—H5B	107.7	O2—C14—H14C	109.5
C5—C6—C7	112.73 (12)	H14A—C14—H14C	109.5
C5—C6—H6A	109.0	H14B—C14—H14C	109.5
C7—C6—H6A	109.0	O3—C15—O4	124.48 (12)
C5—C6—H6B	109.0	O3—C15—C3	124.24 (12)
C7—C6—H6B	109.0	O4—C15—C3	111.20 (11)
H6A—C6—H6B	107.8	O4—C16—H16A	109.5
C7A—C7—C6	112.15 (12)	O4—C16—H16B	109.5
C7A—C7—H7A	109.2	H16A—C16—H16B	109.5
C6—C7—H7A	109.2	O4—C16—H16C	109.5
C7A—C7—H7B	109.2	H16A—C16—H16C	109.5
C6—C7—H7B	109.2	H16B—C16—H16C	109.5
H7A—C7—H7B	107.9	N4—C17—C18	111.88 (12)
C12B—C7A—N8	109.40 (12)	N4—C17—H17A	109.2
C12B—C7A—C7	129.87 (12)	C18—C17—H17A	109.2
N8—C7A—C7	120.47 (13)	N4—C17—H17B	109.2
C8A—N8—C7A	109.31 (12)	C18—C17—H17B	109.2
C8A—N8—H8	126.1 (10)	H17A—C17—H17B	107.9
C7A—N8—H8	123.8 (10)	C17—C18—H18A	109.5
N8—C8A—C9	130.12 (13)	C17—C18—H18B	109.5
N8—C8A—C12A	107.45 (11)	H18A—C18—H18B	109.5
C9—C8A—C12A	122.43 (14)	C17—C18—H18C	109.5
C10—C9—C8A	117.42 (13)	H18A—C18—H18C	109.5
C10—C9—H9	121.3	H18B—C18—H18C	109.5
			
C12B—C1—C2—C3	89.83 (16)	C9—C8A—C12A—C12	−0.5 (2)
C12B—C1—C2—C13	−91.39 (15)	N8—C8A—C12A—C12B	0.01 (15)
C13—C2—C3—N4	161.42 (12)	C9—C8A—C12A—C12B	−179.83 (13)
C1—C2—C3—N4	−19.8 (2)	N8—C7A—C12B—C12A	−1.40 (15)
C13—C2—C3—C15	−16.67 (18)	C7—C7A—C12B—C12A	−175.38 (14)
C1—C2—C3—C15	162.15 (12)	N8—C7A—C12B—C1	177.49 (12)
C2—C3—N4—C17	152.39 (13)	C7—C7A—C12B—C1	3.5 (2)
C15—C3—N4—C17	−29.46 (17)	C12—C12A—C12B—C7A	−178.33 (15)
C2—C3—N4—C5	−70.79 (17)	C8A—C12A—C12B—C7A	0.85 (15)
C15—C3—N4—C5	107.36 (13)	C12—C12A—C12B—C1	2.8 (3)
C3—N4—C5—C6	141.33 (12)	C8A—C12A—C12B—C1	−178.00 (13)
C17—N4—C5—C6	−79.77 (14)	C2—C1—C12B—C7A	−96.28 (16)
N4—C5—C6—C7	−59.93 (15)	C2—C1—C12B—C12A	82.37 (18)
C5—C6—C7—C7A	−53.56 (15)	C14—O2—C13—O1	−2.25 (17)
C6—C7—C7A—C12B	91.06 (18)	C14—O2—C13—C2	175.79 (10)
C6—C7—C7A—N8	−82.35 (15)	C3—C2—C13—O1	−21.88 (18)
C12B—C7A—N8—C8A	1.44 (16)	C1—C2—C13—O1	159.27 (12)
C7—C7A—N8—C8A	176.09 (12)	C3—C2—C13—O2	160.11 (11)
C7A—N8—C8A—C9	178.94 (14)	C1—C2—C13—O2	−18.73 (16)
C7A—N8—C8A—C12A	−0.87 (15)	C16—O4—C15—O3	−9.14 (18)
N8—C8A—C9—C10	−179.83 (14)	C16—O4—C15—C3	174.08 (11)
C12A—C8A—C9—C10	0.0 (2)	C2—C3—C15—O3	124.43 (15)
C8A—C9—C10—C11	0.6 (2)	N4—C3—C15—O3	−53.76 (18)
C9—C10—C11—C12	−0.6 (2)	C2—C3—C15—O4	−58.78 (16)
C10—C11—C12—C12A	0.1 (2)	N4—C3—C15—O4	123.03 (12)
C11—C12—C12A—C8A	0.5 (2)	C3—N4—C17—C18	−62.97 (17)
C11—C12—C12A—C12B	179.58 (15)	C5—N4—C17—C18	159.50 (12)
N8—C8A—C12A—C12	179.34 (12)		

### Hydrogen-bond geometry (Å, º)

**Table:**

*D*—H···*A*	*D*—H	H···*A*	*D*···*A*	*D*—H···*A*
N8—H8···O1^i^	0.891 (17)	2.234 (18)	3.0690 (18)	155.7 (14)
N8—H8···O3^i^	0.891 (17)	2.546 (16)	3.1029 (16)	121.2 (12)

Symmetry code: (i) −*x*+1, *y*+1/2, −*z*+3/2.
